# Sanse Powder Essential Oil Nanoemulsion Negatively Regulates TRPA1 by AMPK/mTOR Signaling in Synovitis: Knee Osteoarthritis Rat Model and Fibroblast-Like Synoviocyte Isolates

**DOI:** 10.1155/2021/4736670

**Published:** 2021-11-28

**Authors:** Mingchao Li, Li Zhang, Zixiu Liu, Li Zhang, Runlin Xing, Songjiang Yin, Xiaochen Li, Nongshan Zhang, Peimin Wang

**Affiliations:** ^1^Department of Orthopedics, The Affiliated Hospital of Nanjing University of Chinese Medicine, Nanjing 210029, China; ^2^Key Laboratory for Metabolic Diseases in Chinese Medicine, First College of Clinical Medicine, Nanjing University of Chinese Medicine, Nanjing 210023, China; ^3^Department of Orthopaedic Surgery, The Third People's Hospital of Kunshan, Suzhou 215300, China 215300; ^4^Jiangsu Province Hospital of Chinese Medicine, Nanjing, Jiangsu 210029, China

## Abstract

Synovitis is the primary driving factor for the occurrence and development of knee osteoarthritis (KOA) and fibroblast-like synoviocytes (FLSs) and plays a crucial role during this process. Our previous works revealed that transient receptor potential ankyrin 1 (TRPA1) ion channels mediate the amplification of KOA synovitis. In recent years, essential oils have been proved to have blocking effect on transient receptor potential channels. Meanwhile, the therapeutic effect of Sanse Powder on KOA synovitis has been confirmed in clinical trials and basic studies; although, the mechanism remains unclear. In the present study, Sanse Powder essential oil nanoemulsion (SP-NEs) was prepared, and then chemical composition, physicochemical properties, and stability were investigated. Besides, both in MIA-induced KOA rats and in LPS-stimulated FLSs, we investigated whether SP-NES could alleviate KOA synovitis by interfering with AMP-activated protein kinase- (AMPK-) mammalian target of rapamycin (mTOR), an energy sensing pathway proved to negatively regulate the TRPA1. Our research shows that the top three substances in SP-NEs were tumerone, delta-cadinene, and Ar-tumerone, which accounted for 51.62% of the total, and should be considered as the main pharmacodynamic ingredient. Less inflammatory cell infiltration and type I collagen deposition were found in the synovial tissue of KOA rats treated with SP-NEs, as well as the downregulated expressions of interleukin (IL)-1*β*, IL-18, and TRPA1. Besides, SP-NEs increased the phosphorylation level of AMPK and decreased the phosphorylation level of mTOR in the KOA model, and SP-NEs also upregulated expressions of peroxisome proliferator-activated receptor-gamma (PPAR*γ*) and PPAR*γ* coactivator-1*α* and downstream signaling molecules of AMPK-mTOR in vivo and in vitro. To conclude, a kind of Chinese herbal medicine for external use which is effective in treating synovitis of KOA was extracted and prepared into essential oil nanoemulsion with stable properties in the present study. It may alleviate synovitis in experimental KOA through the negative regulation of TRPA1 by AMPK-mTOR signaling.

## 1. Introduction

Knee osteoarthritis (KOA) is the most common degenerative joint disease, with joint pain, swelling, and stiffness, affecting nearly 250 million people worldwide [1]. The mechanism of KOA involves all the components of the affected joints and includes joint degeneration, local aseptic inflammation, and peripheral neuropathy, which is complex and has not been fully elucidated. However, researchers have gradually paid attention to the promoting role of synovitis in the occurrence and development of KOA [2, 3]. In our previous study, we have revealed that transient receptor potential ankyrin 1 (TRPA1) of fibroblast-like synoviocytes (FLSs) respond to proinflammatory cytokine stimulation to amplify synovitis in human KOA, indicating the potential of blocking TRPA1 in KOA treatment [4].

TRPA1 is a membrane-associated cation channel highly expressed on FLSs and chondrocytes of KOA animal models [4, 5]. Various exogenous pungent chemicals and proinflammatory agents, as well as endogenous products of oxidative and nitrative stress, can activate TRPA1, leading to an influx of cation ions, in particularly Ca2+, participating multiple physiological responses of the activated cells [6]. Furthermore, TRPA1 is also sensitive to noxious cold and mechanical stimuli, mediating local immune response and pain perception [7]. Interestingly, recent research has revealed the regulation of polyunsaturated fatty acids on TRPA1 [8, 9]. TRPA1 has even been identified as a fatty acid sensor in mammals, suggesting that energy metabolism may highly correlated with TRPA1 activation; although, the precise physiological relevance between the two is still unknown.

Further studies have indicated that the AMP-activated protein kinase (AMPK), a widely expressed intracellular energy sensor, negatively regulates TRPA1[10]. AMPK phosphorylates a broad range of downstream targets, monitoring the balance of cellular energy metabolism [11]. KOA-associated reductions in AMPK activity have been observed both in human chondrocytes and cartilage [12]. Moreover, AMPK regulates mitochondrial function through the mammalian target of rapamycin (mTOR), a critical regulator of cell growth and metabolism, closely associated with inflammation and autophagy in osteoarthritis [13, 14]. Abnormalities in the AMPK-mTOR signaling lead to mitochondrial damage and reactive oxygen species (ROS) accumulation. Metformin, a clinically used AMPK agonist for diabetes, or rapamycin, a specific mTOR inhibitor, inhibits KOA synovial inflammation by increasing phosphorylation of AMPK or inhibiting phosphorylation of mTOR, respectively [14, 15]. In addition, the downstream factors of AMPK-mTOR and peroxisome proliferator-activated receptor-gamma (PPAR*γ*), as well as PPAR*γ* coactivator-1*α* (PGC1*α*), are also closely related to KOA synovitis and local lipid metabolism [16, 17]. Therefore, we speculate that AMPK signaling may alleviate KOA synovitis though the negative regulated TRPA1 by mTOR.

Based on popular use by successive generations, herb medicine has been a source of external pharmacotherapy for KOA treatment [18, 19]. Plants provide multitudinous compounds with potent biological activities which modulate the expression of proinflammatory signals. Notably, recent studies have shown the blocking effect of essential oils (EOs) extracted from plant on transient receptor potential channels [20]. EOs are volatile lipophile liquid or semiliquid, which is a complex mixture of low molecular weight organic compounds produced by plant secondary metabolites. Its main components include monoterpenes and sesquiterpenes, phenylcines, and short chain aliphatic groups. Due to their physicochemical properties (i.e., poor water solubility, scarce stability, high volatility, thermal decomposition, and oxidative degradation), it is difficult to turn EOs into a pharmacological intervention [21, 22]. However, nanotechnological strategies successfully remove the limitations, nanosized EO delivery system, in especial nanoemulsion (NEs), and not only helps in improving the solubility, bioavailability, stability, and pharmacological activity but also improves permeation through the skin and other biological barriers and reach the controlled delivery of active compounds [21, 22].

In our previous study, we analyzed the nonvolatile constituents of Sanse Powder (SP), a Chinese medicine topical patch widely used in the clinical treatment of KOA, and investigated the mechanism of its intervention in KOA pain and synovitis [23]. Ultraperformance liquid chromatography-Q-exactive four-stage pole-orbit well mass spectrometer identified 35 pharmacoactive compounds in SP that may alleviate KOA synovitis and cold pain sensitivity by interfering with metabolic factors in KOA rats [23]. However, whether a stable nanoemulsion can be prepared to help further study of the EOs extracted from SP (SP-EOs), and whether Sanse Powder essential oils in nanoemulsion (SP-NEs) can negatively regulate TRPA1 through AMPK-mTOR signaling to alleviate synovitis of KOA, these all will be discussed in the present study.

## 2. Material and Methods

### 2.1. Experimental Protocol

**Figure 1:**
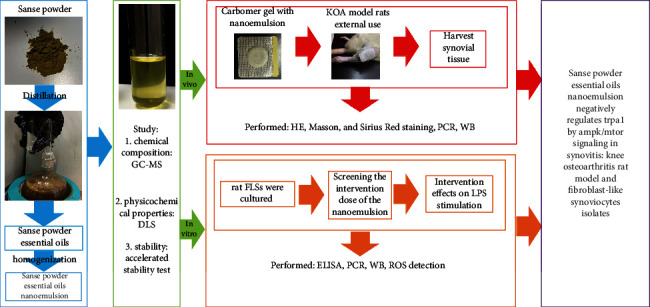
Experimental protocol.

In general, this study includes two parts (Figure 1): (1) preparation and characterization of SP-NEs and (2) study the intervention effect of SP-NEs on KOA synovitis. In the first part, SP-EOs were obtained by distillation and then made into SP-NEs by high pressure homogenization. Next, chemical composition and physical properties were investigated through gas chromatograph-mass spectrometer (GC-MS) and dynamic light scattering (DLS), and stability was tested by a series of static and accelerated methods. In the second part, we loaded SP-NES with Carbomer gel paste externally to KOA model rats or directly to intervene in lipopolysaccharide- (LPS-) stimulated FLSs. The harvested synovial tissue or FLSs were detected by hematoxylin-eosin (HE) staining, masson staining, sirius red staining, polymerase chain reaction (PCR), western blotting (WB), enzyme-linked immunosorbent assay (ELISA), reactive oxygen species (ROS), and other molecular biological detection methods.

### 2.2. Extraction of SP-EOs

Sanse Powder was obtained from Preparation Department, Affiliated Hospital of Nanjing University of Traditional Chinese Medicine, license number: Chinese medicine Z04000566. Briefly, SP-EOs were extracted by the water distillation method. 150 g of Sanse Powder dissolved in 3000 ml of deionized water was boiling using a PTHW type thermoregulated electric heating set device (Nanjing Nan'ao Technology Co., Ltd.) for 5 h. Anhydrous sodium sulfate was added to SP-EOs for dehydration and stored at 4°C in a dark for subsequent experiments.

### 2.3. Preparation of SP-NEs

SP-NEs were prepared employing the method of homogenization [21, 24] using the JN-02HC high-pressure homogenizer (Juneng Biotechnology Co., Ltd., Guangzhou, China). We started with the selection of components for phase A and phase B, and the chosen criteria were whether the sample was clarified or stratified. Caprylic capric triglyceride (GTCC), isopropyl myristic acid, white oil, ethyl oleate, jojoba oil, and olive fruit oil were inspected, as well as the commonly used emulsifiers polysorbate 80 (Tween-80), sorbitan monooleate (Span-80), and coemulsifiers (Octyldodecanol, PPG-26-Buteth-26). Emulsifiers, coemulsifiers, and BHT were mixed and sonicated as the A phase, using a JY92-l ultrasonicator (Xinzhi Biotechnology Co., Ltd., Ningbo, China) at 40 kHz, 5 min. The chosen base oil was heated and stirred until appropriate amount of essential oil was added as the B phase. In the end, the isothermal phase A was slowly added to phase B, and the emulsion mixture was formed by stirring, which was homogenized for 3 times at 120 Pa.

### 2.4. GC-MS Analysis

Chemical composition of SP-EOs and SP-NEs was analyzed on an Agilent 7890A-5975C GC-MS. The column used for the separation was an HP-5 MS capillary column (Thermo Fisher Scientific, Massachusetts, USA). The column was allowed to reach initially a temperature of 45°C for 2 min, then 10°C/min to 100°C for 5 min, and finally to 5°C/min to 200°C for 5 min. The ion source temperature was 230°C, and the inlet temperature was 250°C. Before injection, samples were diluted 1 : 100 in ethyl acetate, and then 1 1 *μ*l was injected in split mode (1 : 20). The peak acquisition was achieved with electron impact (EI, 70 eV) mode in the range 30–500 m/z. Chromatograms obtained were analyzed using Agilent Mass Hunter Qualitative Analysis B.06.00 software and the NIST11 Mass Spectral Search Program, as well as PubChem database.

### 2.5. Characterization of SP-NEs

Visual characterization of SP-NEs was performed by a JEM1230 transmission electron microscope (JEOL, Tokyo, Japan). Particle size measurements, polydispersity index (PDI), and the zeta potential were carried out through DLS analyses by using a NanoZS90 (Malvern Instrument, Malvern, UK). The ultraviolet visible absorption spectrum was obtained using a UV-2401 spectrophotometer (Shimadzu, Tokyo, Japan).

### 2.6. Animals and Treatment Protocol

Forty male SD rats (purchased from Nanjing Qinglongshan Animal Farm, License number: SCXK-SU-201-0001), weighing from 180 to 210 g, were housed in an SPF-grade environment with controlled temperature, humidity, and an alternating 12/12 h light/dark cycle. All animal protocols were approved by the Animal Care and Use Committee of the Nanjing University of Chinese Medicine.

Rats were randomly numbered and divided into four groups: normal, KOA, metformin (purchased from RHAWN, Shanghai, China), and SP-NEs, 10 in each group. The KOA model was induced by intra-articular injection of monoiodoacetic acid (MIA, 2 mg, dissolved in 50 *μ*l sterilized saline), both knees. The other three groups were injected with 50 *μ*l of sterilized saline as control. Next day, the metformin group received metformin (150 mg/kg/d [14, 25]) by oral gavage while the other three groups received isometric sterilized saline as control. The SP-NE group received topical application of SP-NE gel [26] (Carbomer coated with 3% SP-NEs) while the other three groups received Carbomer gel only, for 8 h each day. After 28 days, all animals were sacrificed to harvest the synovial tissue.

### 2.7. FLSs Isolation and Treatment Protocol

FLSs were obtained from additional normal rats. In brief, synovial tissues were minced into pieces of 2-3 mm^2^, digested in 0.1% collagenase type II (Sigma, Missouri, USA) for 1 h. Following cell dissociation, the samples were filtered through a cell strainer. After dissociation, fibroblasts were pelleted by centrifugation at 1500 rpm for 4 min and cultured in DMEM supplemented with 10% fetal bovine serum (Gibco, Thermo Fisher Scientific, Massachusetts, USA).

SP-NEs were sterilized by 0.22 *μ*m filter membrane, and the effect of SP-NEs on FLSs cell viability cells was analyzed by Cell-Counting Kit-8 (Solarbio, Beijing, China). Passages 3-5 of the FLSs were used for the in vitro experiments. Briefly, FLSs in all other groups were stimulated by LPS (5 *μ*g/ml, 12 h) to simulate synovitis except for the normal group. Then, SP-NEs (1 *μ*g/ml, or 4 *μ*g/ml), or the rapamycin (a specific mTOR inhibitor, 500 ng/ml [25, 27], purchased from Med Chem Express, Shanghai, China), were added in different groups for 24 h.

### 2.8. Histological Analysis

Synovial tissues were fixed in 10% neutral formalin after rats executed, embedded in paraffin, and cut into slices, for routine HE staining. Masson staining was performed in turn to potassium dichromate, ferryhematoxylin, bright spring red dye, phosphomolybdic acid, and aniline blue staining after paraffin section was prepared. Sirius Red staining was carried out according to the instructions of Sirius Red Stain Kit (Beyotime Biotechnology, Shanghai, China). Sections were mounted and viewed under a Leica DMI3000B microscope (Leica, Germany), with the use of bright field, or under a Nikon Eclipse E100 microscope (Nikon, Japan), with a polarized light field.

### 2.9. ELISA

The concentration of IL-1*β* and IL-18 in FLSs culture media was determined using a commercially available rat ELISA kit (Nanjing JinYibai Biological Technology Co., Ltd., Nanjing, China) according to the manufacturer's instructions.

### 2.10. Quantitative Real-Time PCR

Total RNA was extracted with TRIzol and assessed by spectrophotometer. Reverse transcription of RNA was performed using Prime Script RT reagent Kit (Vazyme, Nanjing, China). Primer (Sequences as Table 1) was designed and synthesized by Shanghai Biotechnology Service Company. qPCR was performed using Low Rox Plus SYBR Green Master Mix (Yeasen, Shanghai, China) according to manufacturer's instructions, on an ABI PRISM 7300 (Applied Biosystems, California, USA). The mRNA level of individual genes was normalized to GAPDH and calculated by the 2^−*ΔΔ*CT^data analysis method.

### 2.11. Western Blotting

Briefly, synovial or FLSs were mixed with RIPA lysate containing 0.1% PMSF and grinded for 10-15 min. Quantified protein levels were with a BCA protein assay kit (Beyotime Biotechnology, Shanghai, China). Then samples were electrophoresed in SD-PAGE and transferred onto PVDF membrane, blocked with 5% nonfat dry milk for 2 h. The membrane was incubated with first antibody (IL-1*β*, IL-18, PPAR*γ*, PGC1*α*, mTOR, and p-mTOR, 1 : 1000, Cell Signaling Technology, Massachusetts, USA. AMPK*α*, p-AMPK*α*, and TRPA1, 1 : 1000, Affinity Biosciences, Ohio, USA) for overnight at 4°C and then second antibody (1 : 3000, Thermo Fisher Scientific, Shanghai, China) for 2 h. Later, bands were visualized by exposure to the ECL method, and the overall gray value of protein bands was quantified, *β*-actin as internal marker.

### 2.12. Detection of Intracellular ROS

Intracellular ROS were assessed via the probe 2′,7′-dichlorofluorescein-diacetate (DCFH-DA). 10 *μ*M DCFH-DA (Sigma, Missouri, USA) was added to each group of FLSs, incubated at 37°C for 30 min in dark, and then washed with PBS and observed under fluorescence microscope (Leica DMI3000B, Germany).

### 2.13. Statistical Analysis

All experiments were performed independently at least thrice, and data were presented as mean ± standard deviation (SD). Statistical analysis was performed using GraphPad Prism 6.0 Software (California, USA). Group comparisons were assessed with Student's *t*-test or one-way ANOVA for comparison of multiple columns. A value of *P* < 0.05 (two-tailed) was considered as statistically significant. Further statistical differences are expressed as *P* < 0.01.

## 3. Results

### 3.1. Composition Identification of SP-EOs and SP-NEs

**Figure 2:**
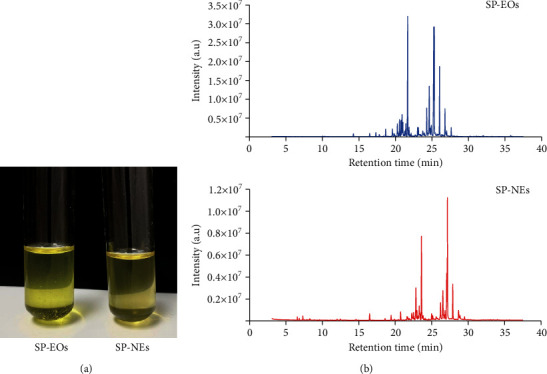
Composition Identification of SP-EOs and SP-NEs. (a) Visual features of SP-EOs and SP-NEs. (b) GS-MS chromatogram of SP-EOs and SP-NEs.

As shown in Figure 2(a), both SP-EOs and SP-NEs were yellowish, clear oils with a distinctive directional scent, and SP-NEs were lighter in color and more uniform in texture. Identified by GC-MS, there were 29 kinds of substances (matching degree > 85%) in SP-EOs, accounting for 88.64% of the total content. Among them, 7 substances with the relative content > 3% accounted for 68.32% of the total content. The top three were Ar-tumerone, delta-cadinene, and tumerone, accounting for 44.73% of the total content (Figure 2(b), Table 2). The formula of SP-NEs was shown in Table 3. 20 kinds of substances (matching degree > 85%) in SP-NEs were identified by GC-MS (Figure 2(b), Table 4), accounting for 97.75% of the total content. There were also 7 substances with the relative content > 3%, and the top 6 kinds were as same as identified in SP-EOs, accounting for 81.10% in total. The top three substances in the list were tumerone, delta-cadinene, and Ar-tumerone, which accounted for 51.62% of the total content, were also as same as identified in SP-EOs, and should be considered as the main pharmacodynamic ingredient.

### 3.2. Physicochemical Characterization of SP-NEs

**Figure 3:**
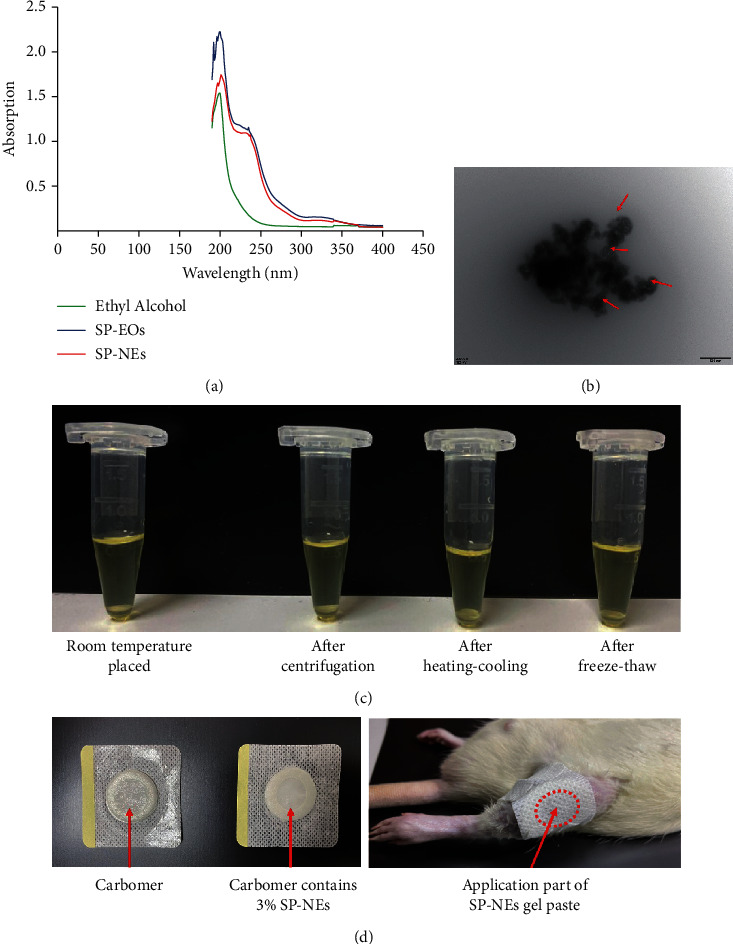
Physicochemical characterization of SP-NEs. (a) Ultraviolet visible spectrum of ethyl alcohol, SP-EOs, and SP-NEs. (b) Transmission electron microscopy observation of SP-NEs, scale bar = 500 nm (×30000). (c) Visual features of SP-NEs after centrifugation, heating–cooling, or freeze-thaw. (d) Carbomer gel with 3% SP-NEs and its external application.

Subsequently, physicochemical characteristics of the prepared SP-NEs were investigated. Ultraviolet spectra (Figure 3(a)) showed that SP-EOs and SP-NEs had highly similar absorption values at 190-400 nm. Transmission electron microscope (Figure 3(b)) showed the evenly dispersed round small particles in SP-NEs. On the completion day of SP-NE preparation (day 0), DLS analysis recorded *Z*-average and PDI values of 20.75 ± 0.17 and 0.29 ± 0.01, respectively. As shown in Table 5, when placed at room temperature for 7 or 14 days, the above two indicators of SP-NEs basically remained unchanged, so does the zeta potential, which presented −13.10 ± 1.75 in day 0. These results proved the thermodynamic stability of the system. And it could also be confirmed by the accelerated stability test, evaluated via centrifugation, heating–cooling cycles, and freeze-thaw cycles. No signs of creaming, phase separation, or cracking were detected after centrifugation, heating–cooling, or freeze-thaw (Figure 3(c)). In order to simulate the clinical application of SP, we further prepared a gel paste containing SP-NEs (Figure 3(d)). Carbomer gel changed from transparent to milky white after loading SP-NEs, and the content of SP-EOs in carbomer gel with 3% SP-NEs was about 2 mg.

### 3.3. SP-NEs Reduce Synovitis and TRPA1 Expression In Vivo

**Figure 4:**
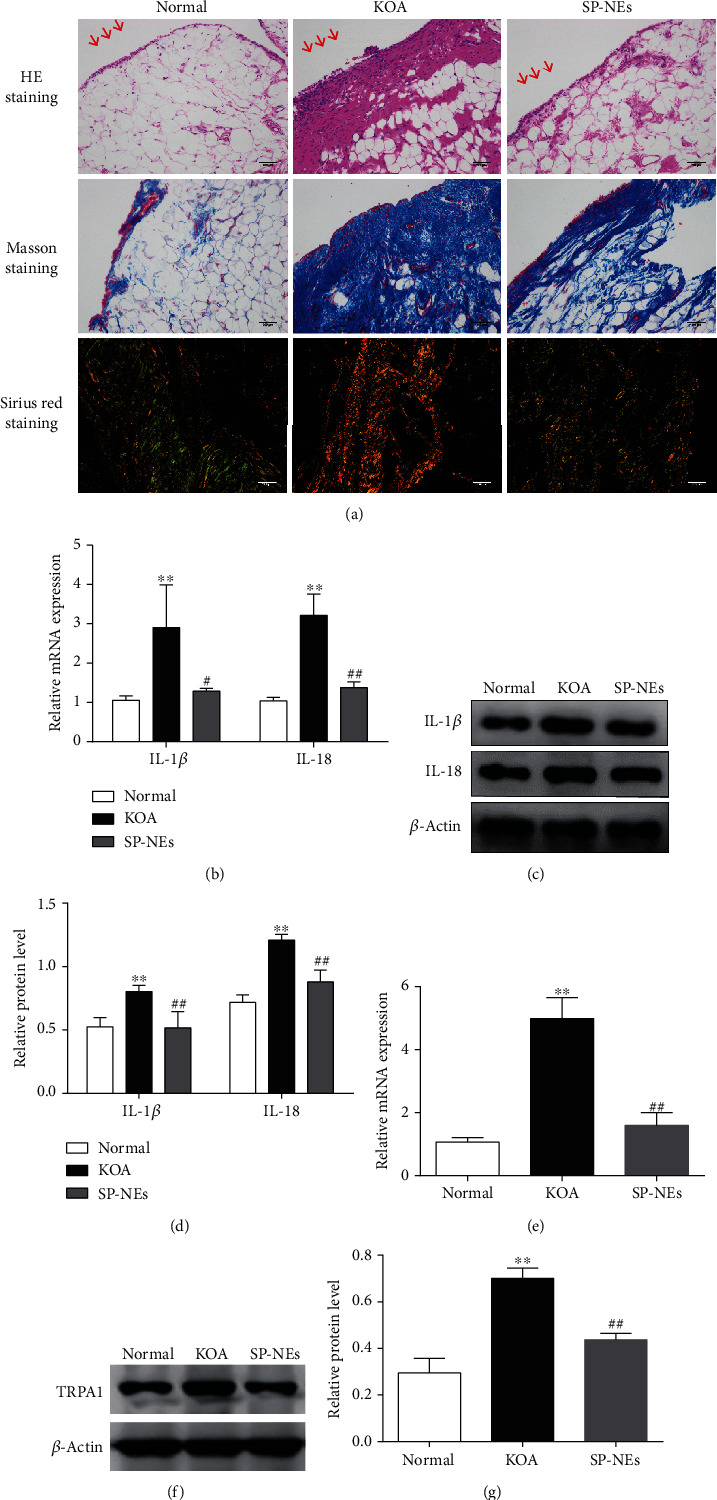
SP-NEs reduce synovitis and TRPA1 expression in vivo. (a). Representative synovial tissues of each group stained with HE, Masson, and Sirius Red staining, ×200, scale bar = 200 *μ*m. Disorderly arranged synovial lining cells, inflammatory cell infiltration (red arrow on HE staining), and increased collagen deposition (blue area in the Masson staining) especially collagen type I (bright yellow area in Sirius Red staining, and the green area represented collagen type III) could be observed in the KOA group. (b). Relative gene expression of IL-1*β* and IL-18 in synovial tissues of rats in each group. ^∗∗^*P* < 0.01 vs. the normal, ^#^*P* < 0.05, ^##^*P* < 0.01 vs. the KOA. (c). Typical protein bands of IL-1*β* and IL-18 for each group. (d). Relative protein level of IL-1*β* and IL-18 in synovial tissues. ^∗∗^*P* < 0.01 vs. the normal, ^##^*P* < 0.01 vs. the KOA. (e). Relative gene expression of TRPA1 in synovial tissues of rats in each group. ^∗∗^*P* < 0.01 vs. the normal, ^##^*P* < 0.01 vs. the KOA. (f). Typical protein bands of TRPA1 (g). Relative protein level of TRPA1 in synovial tissues. ^∗∗^*P* < 0.01 vs. the normal, ^##^*P* < 0.01 vs. the KOA.

To verify the topical effect of SP-NEs on KOA synovitis, rat models of KOA were established by MIA intra-articular injection. As shown in Figure 4(a), HE staining of synovial tissues in the SP-NE group showed an orderly arranged lining of synovial cells, loose connective tissue, and less inflammatory cell infiltration, compared with the KOA group. Decreased collagen deposition (blue area in the Masson staining) especially collagen type I (bright yellow area in Sirius Red staining, while the green area represented collagen type III) could also be observed in the SP-NE group, compared with the KOA group. Besides, both mRNA and protein expressions (Figures 4(b)–4(d)) of IL-1*β* and IL-18 were markedly upregulated in the synovium of KOA rats compared with normal ones (*P* < 0.01). However, the expression levels of these proinflammatory factors in synovium of KOA rats treated with SP-NEs were lower than that of KOA rats (*P* < 0.05). The gene and protein level (Figures 4(e)–4(g)) of TRPA1 showed the same trend (*P* < 0.01).

### 3.4. SP-NEs Regulate mTOR Signaling via Enhanced AMPK Activity In Vivo

**Figure 5:**
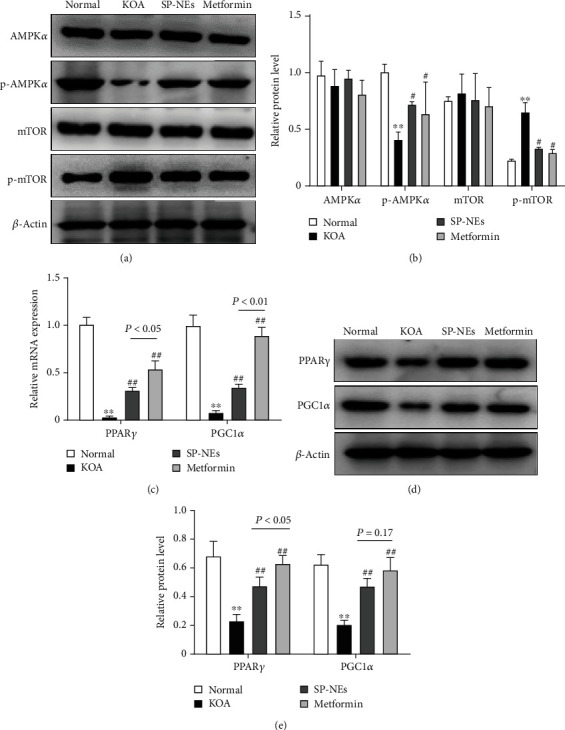
SP-NEs regulate mTOR Signaling via enhanced AMPK activity in vivo. (a) Typical protein bands of AMPK*α*, p-AMPK*α*, mTOR, and p-mTOR in synovium for each group. (b) Relative protein level of AMPK*α*, p-AMPK*α*, mTOR, and p-mTOR for each group. ^∗∗^*P* < 0.01 vs. the normal, ^##^*P* < 0.01 vs. the KOA. (c) Gene level of PPAR*γ* and PGC1*α* in synovial tissues of rats in each group. ^∗∗^*P* < 0.01 vs. the normal, ^##^*P* < 0.01 vs. the KOA, *P* < 0.05, *P* < 0.01, comparison between the underlined two groups. (d) Typical protein bands of PPAR*γ* and PGC1*α* in synovium for each group. (e) Relative protein level of PPAR*γ* and PGC1*α* in synovial tissues of rats in each group. ^∗∗^*P* < 0.01 vs. the normal, ^##^*P* < 0.01 vs. the KOA, *P* < 0.05, *P* = 0.17, comparison between the underlined two groups.

Furthermore, we used metformin, an AMPK signaling agonist with benign intervention in KOA model animals, as a positive control to observe the efficacy of SP-NEs against AMPK-mTOR. In the synovial tissue of KOA rats, there was no difference in the total protein levels of AMPK and mTOR among groups. 150 mg of metformin significantly increased the phosphorylation level of AMPK and decreased the phosphorylation level of mTOR, which were decreased and increased, respectively, compared with the normal group (Figures 5(a) and 5(b)). SP-NEs gel showed comparable efficacy with metformin, and changes in phosphorylation levels of AMPK or mTOR showed no statistically different between the two groups (Figures 5(a) and 5(b)). Besides, the gene and protein expressions of PPAR*γ* and PGC1*α*, downstream signaling molecules of AMPK-mTOR, were lower in the KOA group than normal (Figures 5(c)–5(e)). Both metformin and SP-NEs could increase the expression levels of PPAR*γ* and PGC1*α*, but metformin had a better effect than SP-NEs, and there was a statistical difference between the two.

### 3.5. SP-NEs Modulate AMPK-mTOR Activity in LPS-Stimulated FLSs

**Figure 6:**
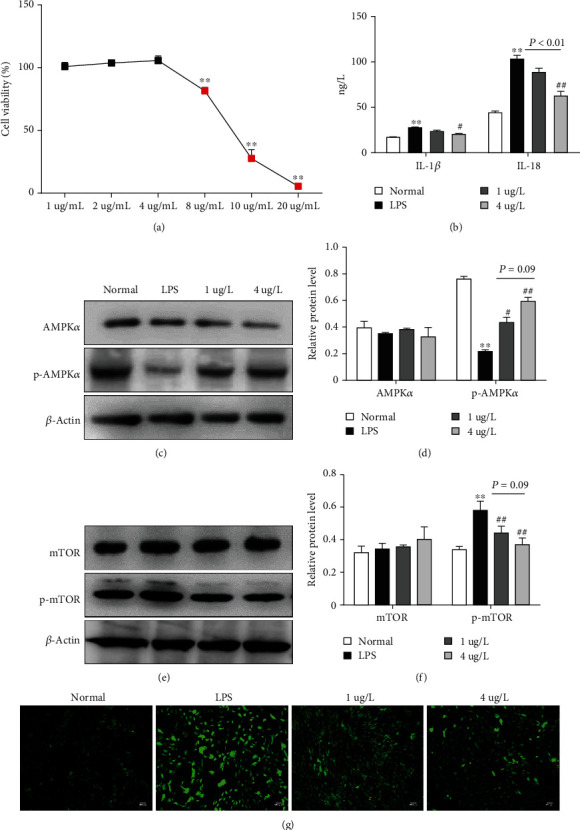
SP-NEs modulate AMPK-mTOR activity in LPS-stimulated FLSs. (a) Different concentrations of SP-NEs on cell viability. (b).The contents of IL-1*β* and IL-18 in the supernatant of FLSs in each group. (c). Typical protein bands of AMPK*α* and p-AMPK*α*, in FLSs for each group. (d) Relative protein level of AMPK*α* and p-AMPK*α* for each group. ^∗∗^*P* < 0.01 vs. the normal, ^#^*P* < 0.05 vs. the KOA, ^##^*P* < 0.01 vs. the KOA, *P* = 0.09, comparison between the underlined two groups. (e) Typical protein bands of mTOR and p-mTOR in FLSs for each group. (f) Relative protein level of mTOR and p-mTOR in each group. ^∗∗^*P* < 0.01 vs. the normal, ^##^*P* < 0.01 vs. the KOA, *P* = 0.09, comparison between the underlined two groups. (g) DCFH-DA probe labeled ROS to reflect mitochondrial function and green fluorescence intensity indicated ROS level.

In the experiment in vitro, the CCK8 method was used to screen the appropriate concentration of SP-NEs, and 1 *μ*g/ml and 4 *μ*g/ml were determined as the high dose and low dose, respectively (Figure 6(a)). 5 *μ*g/ml LPS stimulation could significantly increase the content of IL-1*β* and IL-18 in the supernatant of FLSs, and SP-NE intervention at 4 *μ*g/ml could effectively reduce the content of both proinflammatory factors, while 1 *μ*g/ml can only reduce IL-18 (Figure 6(b)). Besides, either 4 *μ*g/ml or 1 *μ*g/ml of SP-NEs could increase AMPK phosphorylation which were downregulated after LPS stimulation and decrease mTOR phosphorylation which were upregulated after LPS stimulation, but there was no difference between the two concentrations (Figures 6(c)–6(f)). Furthermore, DCFH-DA fluorescent probe was used to measure mitochondrial function. DCFH-DA freely passed through the cell membrane and oxidized by intracellular reactive oxygen species (ROS), showing green fluorescence. As shown in Figure 6(g), LPS induced ROS accumulation in FLSs, while 4 *μ*g/ml or 1 *μ*g/ml of SP-NEs could reduce the increased fluorescence intensity of LPS.

### 3.6. SP-NEs Suppress TRPA1 though mTOR Signaling in LPS-Stimulated FLSs

Since the dose of SP-NEs at 1 *μ*g/ml or 4 *μ*g/ml showed no statistical difference in the phosphorylation levels of AMPK and mTOR in FLSs, 4 *μ*g/ml was selected in the subsequent experiment. We used rapamycin, an mTOR inhibitor, as a control to investigate whether SP-NEs could affect the expression of TRPA1 by blocking mTOR.

**Figure 7:**
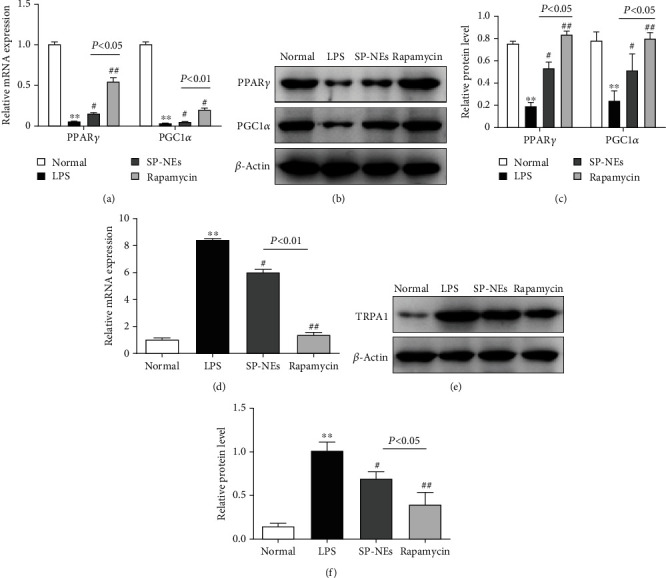
SP-NEs suppress TRPA1 though mTOR in LPS-stimulated FLSs. (a) Gene level of PPAR*γ* and PGC1*α* in FLSs of each group. ^∗∗^*P* < 0.01 vs. the normal, ^#^*P* < 0.05, ^##^*P* < 0.01 vs. the LPS, *P* < 0.05, *P* < 0.01, comparison between the underlined two groups. (b) Typical protein bands of PPAR*γ* and PGC1*α*, in FLSs for each group. (c) Relative protein level of PPAR*γ* and PGC1*α* for each group. ^∗∗^*P* < 0.01 vs. the normal, ^#^*P* < 0.05, ^##^*P* < 0.01 vs. the LPS, *P* < 0.05, comparison between the underlined two groups. (d). Gene level of TRPA1 in FLSs of each group. ^∗∗^*P* < 0.01 vs. the normal, ^#^*P* < 0.05, ^##^*P* < 0.01 vs. the LPS, *P* < 0.01, comparison between the underlined two groups. (e). Typical protein bands of TRPA1, in FLSs for each group. (f). Relative protein level of TRPA1 for each group. ^∗∗^*P* < 0.01 vs. the normal, ^#^*P* < 0.05, ^##^*P* < 0.01 vs. the LPS, *P* < 0.05, comparison between the underlined two groups.

Both 500 ng/ml rapamycin and 4 *μ*g/ml SP-NEs (Figures 7(a)–7(c)) significantly increased the expressions of PPAR*γ* and PGC1*α* after LPS challenge (*P* < 0.05), while rapamycin further amplified the upregulation compared with SP-NEs, and the difference was statistically significant (*P* < 0.05). At the same time, the expression of TRPA1 (Figures 7(d)–7(f)) was downregulated after mTOR was blocked by rapamycin (*P* < 0.01), and SP-NEs could achieve the same effect (*P* < 0.05); although, the downregulated degree was still significantly lower than rapamycin (*P* < 0.05).

## 4. Discussion

In the present study, we first extracted the EOs from Sanse Powder, a Chinese herbal compound prescription for external use which is effective in clinical treatment of KOA and studied the preparation process of transforming them into nanoemulsion. Consistent with the other scholars [28, 29], we also screened the matrix materials and proportions needed for SP-NES through high-pressure homogenization method. The obtained SP-NEs observed as uniformly dispersed small particles under transmission electron microscopy. DLS analysis recorded *Z*-average and PDI values of 20.75 ± 0.17 and 0.29 ± 0.01, respectively. The *Z*-average value used in DLS presents the cumulant mean, that can be defined as the harmonic intensity averaged particle diameter. Assuming that the particle population is a simple Gaussian distribution, the *Z*−average is the mean, and the PDI is related to the width of this simple distribution [21]. When PDI is less than 0.3, the system has better dispersion and less adhesion and aggregation, and when PDI is approximately 0, the nanocarrier is closer to a monodisperse system. Besides, zeta potential of SP-NEs presented −13.10 ± 1.75 mV, and it is well known that when the absolute value of zeta potential is between 5 and 20 mV; the nanoemulsion shows short-term stability [30].

Subsequently, we compared the composition of SP-NES and SP-EOS, and both ultraviolet visible spectrum and GC-MS showed that the main components and contents of the two are highly consistent. Chemically, EOs are natural mixtures with complex compositions, but 2-3 major components are present in fairly high concentrations (20%-70%), while others are present in trace amounts [31, 32]. Single herbal EOs are combined with the above rules, as are compound herbal EOs. Thus, the top three active ingredients identified in SP-EOs should be considered as the main pharmacodynamic ingredient, which were tumerone, delta-cadinene, and Ar-tumerone, accounted for 51.62% of the total content. Notably, both tumerone and Ar-tumerone have been reported to have anti-inflammatory effects [33, 34] and may be extracted together as the main pharmacological substance of *Curcuma longa* Radix [35]. Corresponding to the Sanse Powder, we speculate that tumerone and Ar-tumerone may be derived from Pian Jiang Huang, a traditional Chinese medicine from the *Curcuma* genus. Furthermore, the encapsulation of curcumin nanoemulsion has always been an interesting topic for researchers [36]. In addition, the efficacy of *Curcuma* and its extract in the clinical treatment of KOA has been supported by a large number of studies [37, 38]. In vitro studies demonstrated that curcumin could prevent the apoptosis of chondrocytes and suppress the release of proteoglycans and metal metalloproteases and inflammatory cytokines in KOA [39]. The above reasons all confirmed the potential value of further exploring the efficacy targets of SP-NEs in the treatment of KOA.

Lv et al.'s study revealed that a curcumin derivative could negative regulate AMPK on TRPA1 in vivo and in vitro [39]. Similar to this, we also confirmed in this study that SP-NEs could reduce KOA synovitis via negative regulation of TRPA1 by AMPK-mTOR signaling. In this process, the in vivo and in vitro models of KOA were constructed by intra-articular injection with MIA 2 mg or LPS challenge at 5 *μ*g/ml for 12 h, respectively. MIA causes cytotoxicity to chondrocytes and induces apoptosis, resulting in cartilage damage that not only mimics the development of human KOA but also the inflammatory environment of KOA [40]. Therefore, MIA or LPS was used for the same purpose: to simulate the KOA inflammatory environment. This approach has also been reported in other studies [41, 42]. AMPK-mTOR signals are central regulators of cellular metabolism and triggered by metabolic cues such as amino acid sufficiency or oxidative stress [43]. Thus, an insufficient phosphorylation level of AMPK leads to mTOR activation. As a serine/threonine kinase, mTOR phosphorylates four signature substrates: ribosomal S6 kinase, eukaryotic translation initiation factor 4E-binding protein 1, signal transducer and activator of transcription 3, and AMBRA1. In turn, activation of mTOR forms two distinct complexes, mTORC1 and mTORC2, and executes cell-type-specific commands for growth, proliferation, and survival [43, 44]. Accumulating evidence highlights the vital role of mTOR in the pathogenesis and progression of OA, while PPAR*γ* and PGC1*α* are two major participants downstream of mTOR [16, 17, 25, 27]. PPAR*γ* is a transcriptional coactivator that binds to a diverse range of transcription factors. Together with PGC1*α*, they play a key part in the regulation of the metabolism and consequently modulating the production of ROS, autophagy, and mitochondrial biogenesis.

In animal experiments, the topical application of SP-NEs gel paste can effectively reduce synovitis of KOA rats and downregulate the gene and protein expressions of IL-1*β*, IL-18, and TRPA1. Besides, SP-NEs showed a benign intervention effect on AMPK-mTOR signaling in vivo and in vitro, and the efficacy of some targets was comparable to that of metformin. At last, we investigated the correlation between AMPK-mTOR and TRPA1 with rapamycin as a control. Rapamycin is an allosteric inhibitor of mTOR that acts by forming a high-affinity complex with the intracellular protein FKBP12, primarily affect mTORC1, and might cause a secondary activation of mTORC2 [15, 27, 45]. In FLSs, rapamycin significantly upregulated the reduced expression of PPAR*γ* and PGC1*α* induced by LPS stimulation and also reduced the upregulation of TRPA1, while SP-NEs represented a similar effect to rapamycin, but showed a weaker regulation effect than rapamycin. Above all, we believe that the role of SP-NEs in alleviating synovitis of KOA is related to its intervention in AMPK-mTOR signaling and subsequent negative regulation of TRPA1.

## 5. Conclusions

In summary, in the present study, a kind of Chinese herbal medicine for external use which is effective in treating synovitis of KOA was extracted and prepared into essential oils nanoemulsion with stable properties and may alleviate synovitis in experimental KOA through the negative regulation of TRPA1 by AMPK-mTOR signaling.

## Figures and Tables

**Table 1:** Nucleotide sequences of primers.

Target	Forward primer (5′-3′)	Reverse primer (5′-3′)
IL-1*β*	ATGGTCGGGACATAGTTGA	CTTGGCAGAGGACAAAGG
IL-18	AACGAATCCCAGACCAGAC	AGAGGGTAGACATCCTTCCAT
TRPA1	GAATTTCCAAGATGCCTTCAG	CGGTAATTGATGTCTCCCAG
PPAR*γ*	CCTCGAGGACACCGGAGA	CACGGAGCTGATCCCAAAGT
PGC1*α*	GTAGATCCTCTTCAAGATCCTG	CATACAAGGGAGAATTGCGA
GAPDH	GTTGTGGCTCTGACATGCT	CCCACGGATGCCCTTTAGT

**Table 2:** Major chemical composition of SP-EOs.

Ranking	Content (%)	Component	CAS number	Formula	Match degree
1	17.3669	Ar-tumerone	1000292-71-0	C_15_H_20_O	97
2	15.5457	(+)-Delta-cadinene	000483-76-1	C_15_H_24_	98
3	11.8154	Tumerone	180315-67-7	C_15_H_22_O	90
4	7.9372	Curlone	087440-60-6	C_15_H_22_O	95
5	7.9107	.Tau.-cadinol	005937-11-1	C_15_H_26_O	86
6	3.9382	(-)-Zingiberene	000495-60-3	C_15_H_24_	94
7	3.8023	Transligustilide	1000365-98-8	C_12_H_14_O_2_	94

**Table 3:** Formulation of SP-NEs.

Ingredient of A phase	Ratios (%)	Ingredient of B phase	Ratios (%)
Tween-80	50		
Span-80	5	GTCC	10
Octyldodecanol	12.5	Sanse Powder Essential Oils	20
PPG-26-Buteth-26	2		
BHT	0.5		

**Table 4:** Major chemical composition of SP-NEs.

Ranking	Content (%)	Component	CAS number	Formula	Match degree
1	24.2173	Tumerone	180315-67-7	C_15_H_22_O	96
2	17.6219	(+)-Delta-cadinene	000483-76-1	C_15_H_24_	97
3	9.7825	Ar-tumerone	180315-67-7	C_15_H_20_O	97
4	8.7263	.Tau.-cadinol	005937-11-1	C_15_H_26_O	87
5	8.7192	(-)-Zingiberene	000495-60-3	C_15_H_24_	94
6	7.2374	Curlone	087440-60-6	C_15_H_22_O	97
7	4.7994	Cadinadiene-1,4	016728-99-7	C_15_H_24_	93

**Table 5:** Stability evaluation of SP-NEs.

Time points	*Z*-average (nm)	Zeta potential (mV)	PDI
Day 0	20.75 ± 0.17	−13.10 ± 1.75	0.29 ± 0.01
Day 7	20.47 ± 0.16	−12.80 ± 1.08	0.26 ± 0.01
Day 14	19.71 ± 0.30	−12.63 ± 0.50	0.18 ± 0.02

## Data Availability

The data used to support the findings of this study are available from the corresponding author upon request.

## Abbreviations

KOA: Knee osteoarthritis
TRPA1: Transient receptor potential ankyrin 1
FLSs: Fibroblast-like synoviocytes
AMPK: AMP-activated protein kinase
mTOR: Mammalian target of rapamycin
PPAR*γ*: Peroxisome proliferator-activated receptor-gamma
PGC1*α*: PPAR*γ* coactivator-1*α*
EOs: Essential oils
NEs: Nanoemulsion
SP: Sanse Powder
SP-EOs: Essential oils extracted from Sanse Powder
SP-NEs: Sanse Powder essential oils in nanoemulsion
GC-MS: Gas chromatograph-mass spectrometer
PDI: Polydispersity index
DLS: Dynamic light scattering.
